# Non‐Surgical Treatment of Bilateral Dens Invaginatus in Maxillary Lateral Incisors: A Case Report

**DOI:** 10.1002/ccr3.70482

**Published:** 2025-05-12

**Authors:** Ali Chamani, Maryam Forghani, Ghazal Asadi

**Affiliations:** ^1^ Dental Materials Research Centre, Faculty of Dentistry Mashhad University of Medical Sciences Mashhad Iran; ^2^ Dental Research Centre, Faculty of Dentistry Mashhad University of Medical Sciences Mashhad Iran; ^3^ Student Research Committee, Faculty of Dentistry Mashhad University of Medical Sciences Mashhad Iran

**Keywords:** cone‐ beam computed tomographic imaging, dens in dente, dens invagination, non‐surgical endodontic treatment, periapical lesion

## Abstract

Dens invaginatus (DI) is a dental developmental malformation, with 43% of the reported cases involving bilateral occurrence. Based on its manifestations, DI is classified into different types. Due to the complex anatomy, teeth with DI are more susceptible to caries and pulpitis and are commonly associated with incomplete root formation. The correct diagnosis in these cases requires a combination of clinical examinations, pulp sensitivity tests, and radiographic examinations. The treatment plan may vary from a simple conservative sealing of the invagination to extraction of the involved tooth, depending on the extent of the tooth involvement and severity of the condition. This article reports a case of type II bilateral invagination of two maxillary lateral incisors, the maxillary left lateral incisor with necrotic pulp associated with a large periapical lesion and the maxillary right lateral incisor with reversible pulpitis. Each tooth was successfully treated with different non‐surgical methods based on the extent of its involvement. At the follow‐ups, the patient was asymptomatic with no signs or symptoms of inflammation, and the two‐year follow‐up showed significant healing of both teeth, validating the success of the treatment. The results of this case emphasize the effectiveness of non‐surgical treatment methods when applied appropriately, as well as the importance of early detection in the successful management of teeth with DI.

**Summary**

- Early treatment of dens invaginatus (DI) helps eliminate infections and prevent pulp exposure, that could result in abscess formation and subsequent tooth loss.
- Early intervention helps maintain pulp vitality, ensures proper tooth development, and reduces the likelihood of requiring complex treatment procedures in the future.

## 1 Introduction

Dens Invaginatus (DI) or dens in dente is a relatively prevalent condition, affecting up to 10% of teeth [1, 2]. It is a developmental dental malformation resulting from the invagination of the enamel organ into the dental papilla before the calcification during odontogenesis of the soft tissue. As the hard tissues are formed, the invaginated tissue forms a small tooth within the future pulp chamber. In radiographic images, DI appears like a tooth inside another tooth, which can be limited to the crown or extended to the root [2, 3, 4].

DI can affect any tooth, but it mainly occurs in premolars [5]. 43% of the reported cases involve bilateral occurrence of the contralateral teeth [2]. Several factors can explain the etiology of this abnormality, such as growth failure, abnormal proliferation, distortion of the enamel organ, genetics, nutritional factors, infections, and external trauma. It is also suggested that more than one factor may contribute to the occurrence of this malformation [1, 6, 7, 8].

Based on the vertical extension of the invagination, DI has been categorized into three types, each varying in the extent of invagination and required management approaches. Type I is an invagination limited to the tooth crown, extending up to the cementoenamel junction (CEJ). In Type II, the invagination extends beyond CEJ into the root, with no communication to the periodontium. In type III, the invagination opens up to the periodontal ligament and forms an additional lateral or apical foramen. Types I and II are considered incomplete invaginations, while Type III is classified as complete invagination [2, 4, 9].

DI is often afflicted with pulp and periradicular disease due to debris accumulation within its complex anatomy [10, 11]. In most cases, endodontic treatment is required, which is often considered challenging due to the complex and variant anatomy [12]. Management of DI varies in different cases, and an anatomy‐based treatment plan with consideration of tooth and tissue involvement is essential for successful outcomes [1, 3]. In cases with pulp involvement and periradicular inflammatory lesions, more complicated approaches are required [2, 13]. If the pulp responds normally to sensitivity tests, sealing the invagination or performing a minimal restoration is recommended to prevent bacterial penetration [14, 15, 16].

This article presents a case of bilateral Type II DI in the maxillary lateral incisors, with one tooth exhibiting pulp necrosis and periapical inflammation, and the other showing mild pulp involvement with intact surrounding tissues. For each tooth, a combination of radiographic images and pulp testing was used to ensure accurate diagnosis and treatment planning.

## 2 Case History

A 16‐year‐old female patient was referred to the endodontic department of the faculty of dentistry at Mashhad University of Medical Sciences with a history of repeated swelling in the area of the maxillary anterior teeth. The patient's medical history revealed no significant systemic conditions that would contraindicate endodontic treatment.

## 3 Examination and Diagnosis

**FIGURE 1:**
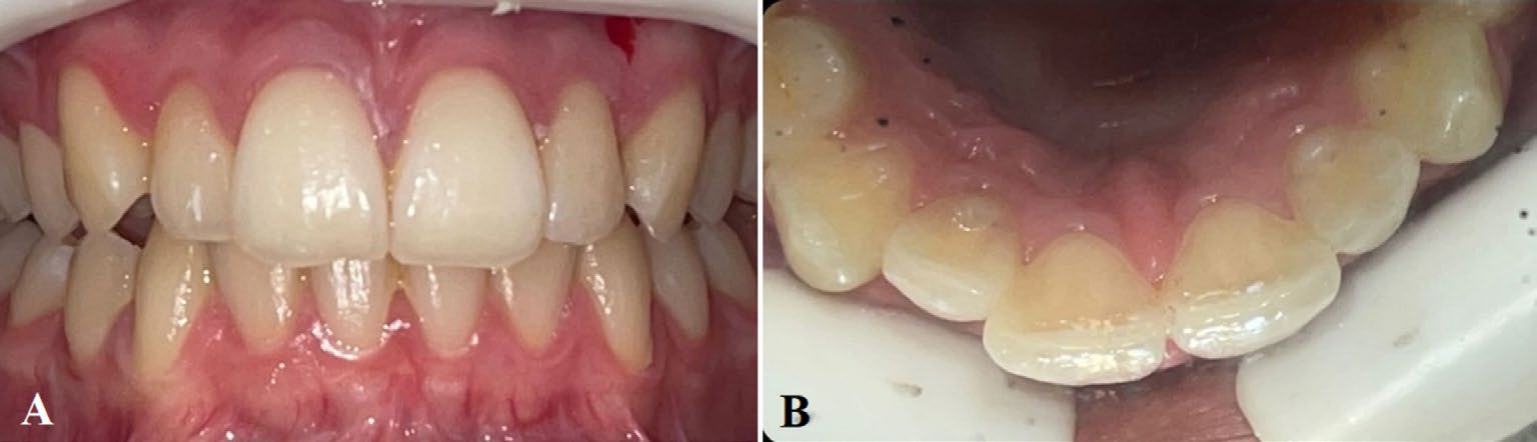
(A, B) Clinical images of maxillary lateral incisors with DI.

**FIGURE 2:**
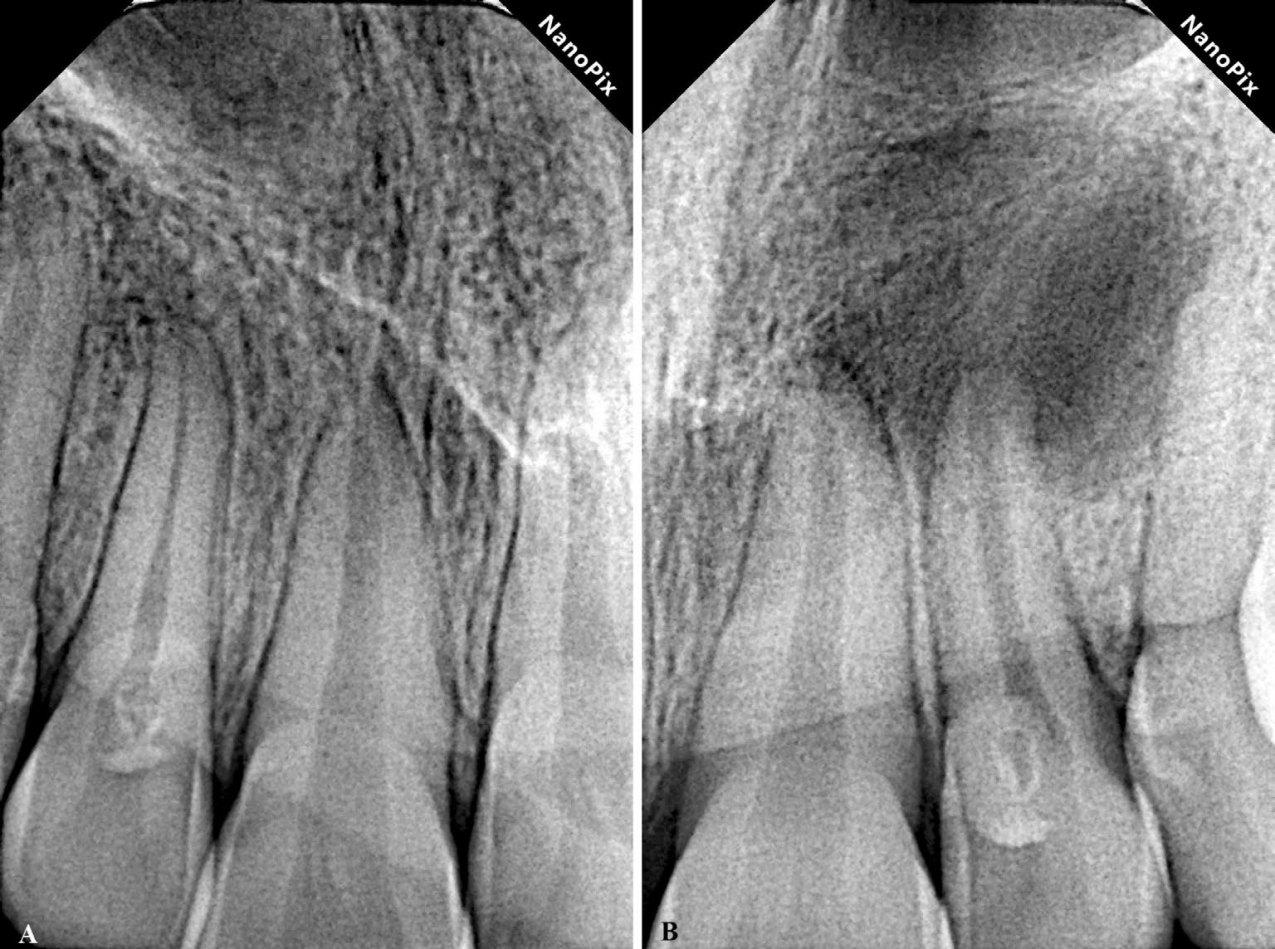
(A) Periapical Radiographic image showing the right maxillary lateral incisor (tooth #7) with DI. (B) Periapical Radiographic image showing the left maxillary lateral incisor (tooth #10) with DI, open apex, and an extensive periapical radiolucency.

The clinical and radiographic examinations revealed bilateral type II DI of maxillary lateral incisors (teeth #7 and #10) (Figures 1 and 2). A thorough periodontal evaluation and clinical testing (pulp and periapical tests) were used to determine the preliminary diagnosis on both teeth. The maxillary right lateral incisor (tooth #7) was responsive to pulp sensitivity testing. Responses to percussion and palpation were normal, and radiographically, there was no evidence of osseous changes (Figure 2A). The maxillary right lateral incisor (tooth #7) was diagnosed with reversible pulpitis and normal apical tissue.

The Maxillary left lateral incisor (tooth #10) showed a large apical radiolucency extending to the floor of the nasal cavity. Moreover, the periapical radiographic image of tooth #10 showed a wide‐open apical foramen (Figure 2B). Periodontal probing depths were all within normal limits. Tooth #10 did not respond to thermal (cold and heat) and electric pulp tests. There was no percussion or palpation in the region. The maxillary left lateral incisor (tooth #10) was diagnosed with pulp necrosis and chronic apical abscess.

**FIGURE 3:**
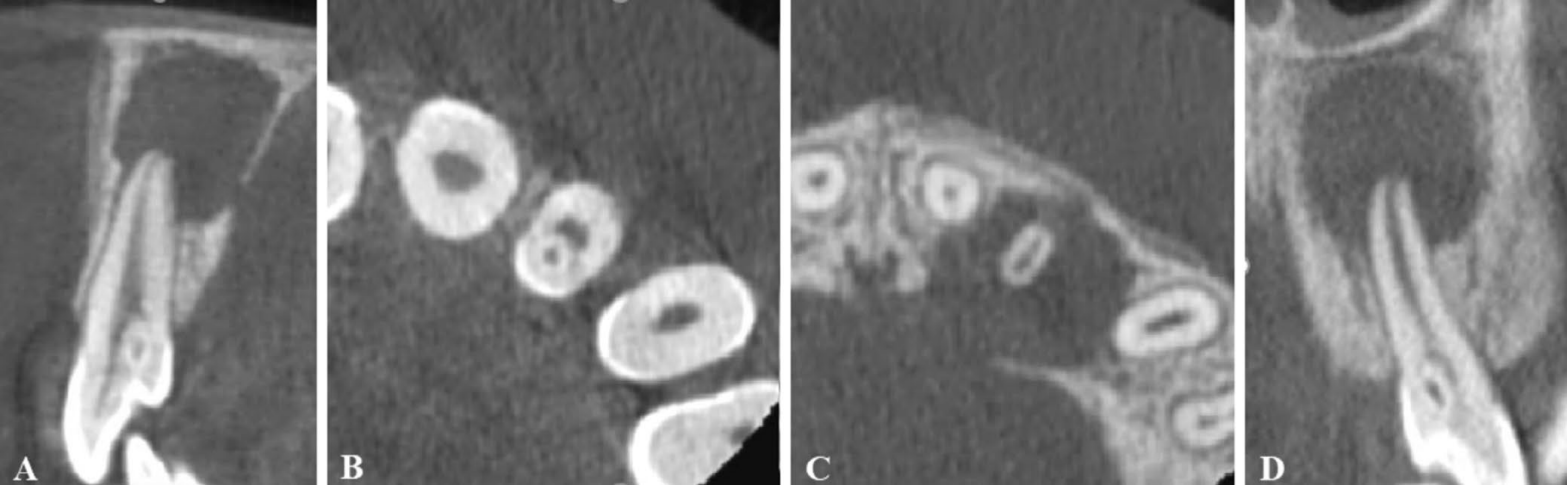
(A) The sagittal CBCT sections of the maxillary left lateral incisor with DI types II. (B) The axial CBCT section shows the invagination of the maxillary left lateral incisor lined by enamel. (C) The axial CBCT section shows the perforation of the palatal cortex due to the extensive periapical lesion of the maxillary left lateral incisor. (D) The coronal CBCT sections of the maxillary left lateral incisor with DI types II, open apex, and an extensive periapical radiolucency.

**FIGURE 4:**
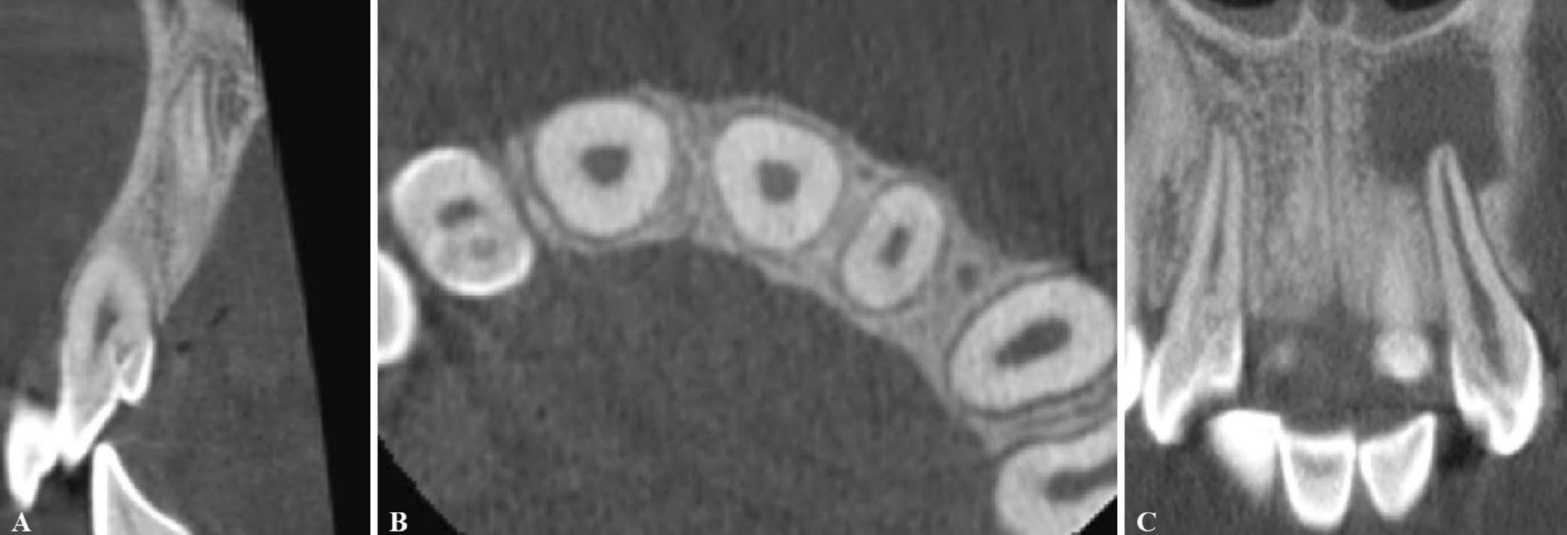
(A) The sagittal CBCT section shows the maxillary right lateral incisor with DI. (B) The axial CBCT section shows the invagination of the maxillary right lateral incisor. (C) The coronal CBCT sections of the maxillary right lateral incisor (tooth #7) with DI and maxillary left incisor (tooth #10) with DI and an extensive periapical radiolucency.

Given the unique anatomy of teeth with DI, cone‐beam computed tomography (CBCT) was ordered to further evaluate the condition of the teeth and surrounding tissues. The patient was informed of the intended benefits and potential risks of the CBCT scan. After obtaining written informed consent, a CBCT of teeth #7 and #10 was performed (Figures 3 and 4). Examination of cross‐sectional images of tooth #10 revealed the perforation of the palatal cortex in the region of tooth #10 due to the extent of the periapical lesion (Figure 3A,C).

## 4 Treatment

Based on the clinical and radiographic findings, the treatment plan for tooth #10 involved non‐surgical endodontic treatment due to pulp necrosis and the extensive periapical lesion. For tooth #7, a conservative restorative procedure with composite resin was planned to seal the invagination. Due to the extensive periapical lesion and the open apex, the possibility of additional apical surgery for tooth #10 was considered. After explaining the diagnosis, treatment plan, and potential risks, written informed consent was obtained from the patient.

**FIGURE 5:**
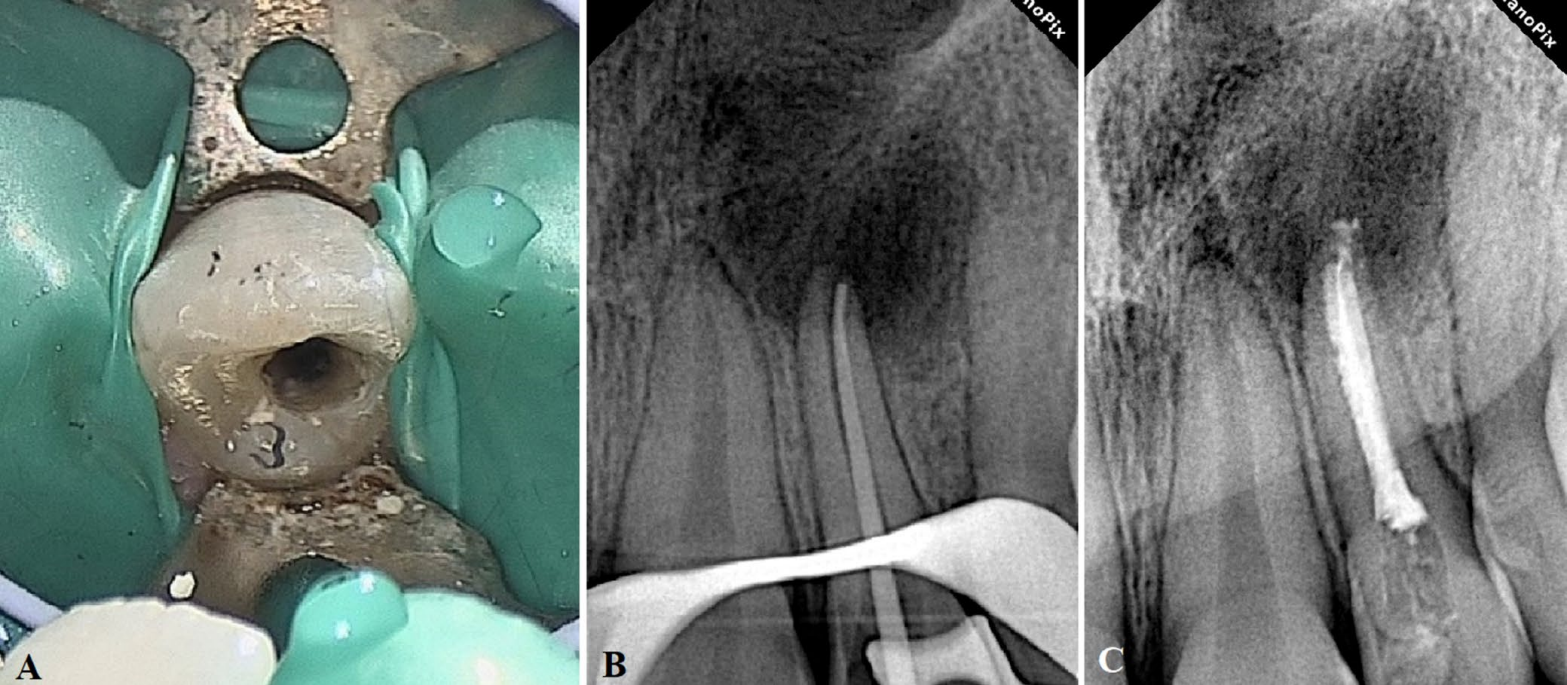
(A) Clinical view of the access cavity of the maxillary left incisor. (B) A periapical radiographic image showing the fitting of the master cone. (C) The postoperative radiograph of the maxillary left incisor.

The endodontic treatment of tooth #10 was started by administering local anesthetics (2% lidocaine and epinephrine 1:100,000) (Daroupakhsh, Tehran, Iran) and rubber dam isolation. The access cavity was prepared using a high‐speed diamond round bur No. 2 (Jota AG, Rüthi, Switzerland) under magnification (Carl Zeiss, Meditec Inc., Dublin, CA, USA) (Figure 5A). After locating the orifice, the working length was estimated using an apex locator (Dempex, DEM Ltd., Barnstaple, Devon, England) and a radiographic image. Chemo‐mechanical debridement was completed by crown‐down technique with K‐files (MANI K‐files, MANI, Japan) up to size #50 and frequent irrigation with 5.25% sodium hypochlorite and sterile saline.

After drying the canal with paper points (META, Chugbuk, South Korea), due to the wide apical foramen (size #80) (Figure 5B), the apical half of the canal was sealed with Cold Ceramic (CC) (SJM, Iran) following the manufacturer's instructions using MAP One carrier (Maillefer, Dentsply, Switzerland) and a size #20 finger plugger (Maillefer, Dentsply, Switzerland). The coronal half of the canal was obturated with gutta‐percha (META, Chugbuk, South Korea) and AH plus sealer (Dentsply DeTrey, Konstanz, Germany) using the warm vertical technique by FastFill warm obturator (Fast Fill Obturation System, Eighteeth, China) (Figure 5C). Cavit (Cavisol, Tehran, Iran) was used as the temporary restoration. The postoperative guidance was given to the patient, including oral hygiene instructions and analgesic medication (Ibuprofen 400 mg) for postoperative symptoms.

The restorative treatment of tooth #7 involved sealing the invagination with flowable composite resin. The procedure began with the administration of local anesthetics (2% lidocaine with epinephrine 1:100,000) (Daroupakhsh, Tehran, Iran) followed by rubber dam isolation. A high‐speed round diamond bur (Jota AG, Rüthi, Switzerland) was used to clean and shape the invagination for restoration. A 35% phosphoric acid etchant solution (Ultra‐Etch, Ultradent, USA) was used according to the manufacturer's instructions to etch and prepare the tooth surface.

After etching and rinsing, the bonding agent (Bonding Saremco 5th generation, SAREMCO Dental AG, Switzerland) was applied to the prepared surface, air‐dried, and light‐cured according to the manufacturer's instructions. The invagination was then filled and sealed with flowable composite resin (Palfique Universal Flow, Tokuyama Dental, Japan) and light‐cured, following the manufacturer's instructions. Finally, the restoration was finished and polished to achieve a smooth surface and proper occlusion.

## 5 Follow‐Up

Due to the extensive periapical lesion and the risk of nasal floor perforation in the event of treatment failure and lesion progression, the patient was monitored during frequent follow‐up visits to evaluate the healing process and assess the potential need for apical surgery. During these follow‐ups, the patient remained asymptomatic, and healing was confirmed with periapical radiographs. After confirming the absence of signs and symptoms, tooth #10 was restored with direct composite resin restoration (Gradia Direct, GC Corporation, Tokyo, Japan).

**FIGURE 6:**
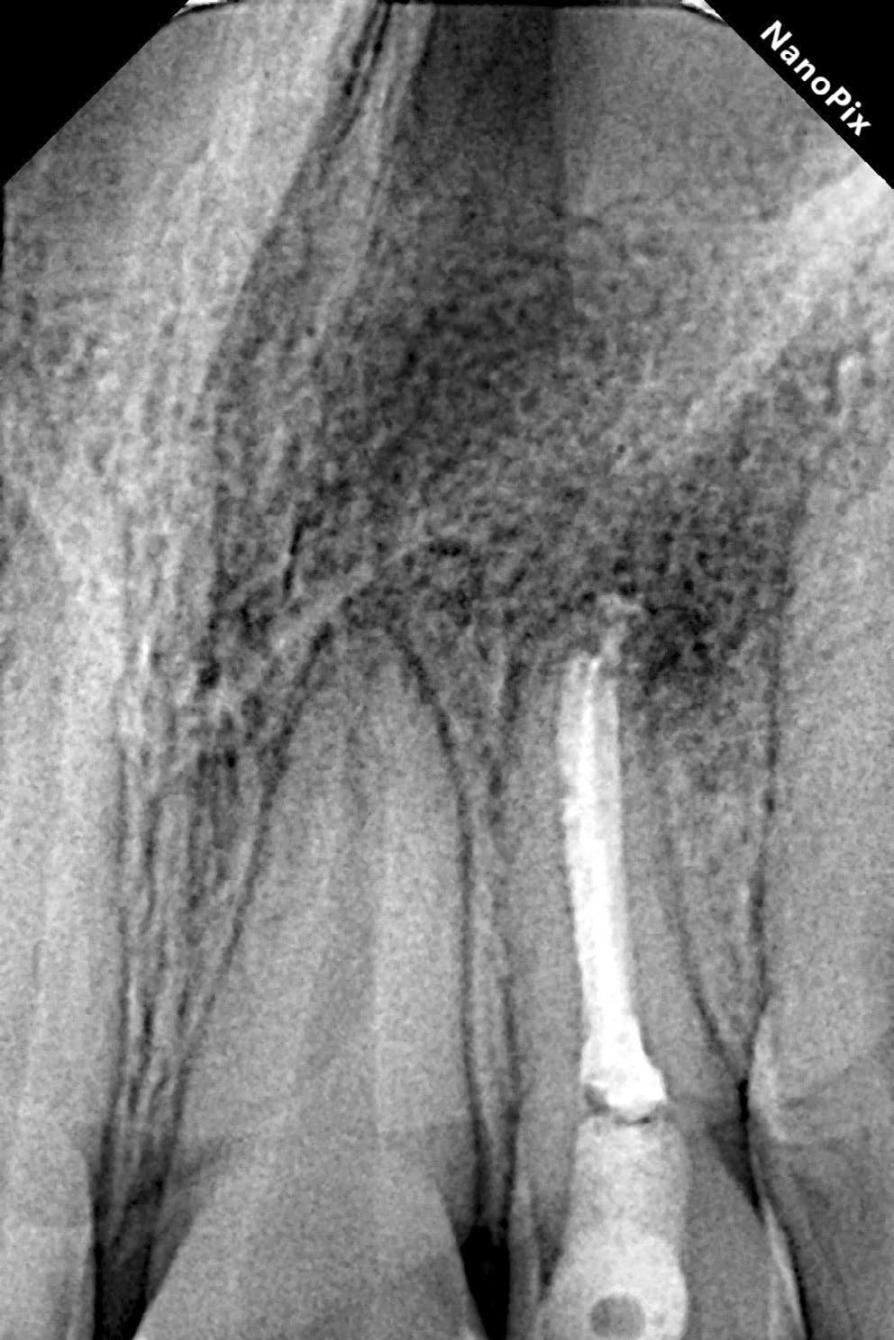
The six‐month follow‐up radiographic image shows favorable healing of the periapical lesions of the maxillary left incisor (tooth #10).

**FIGURE 7:**
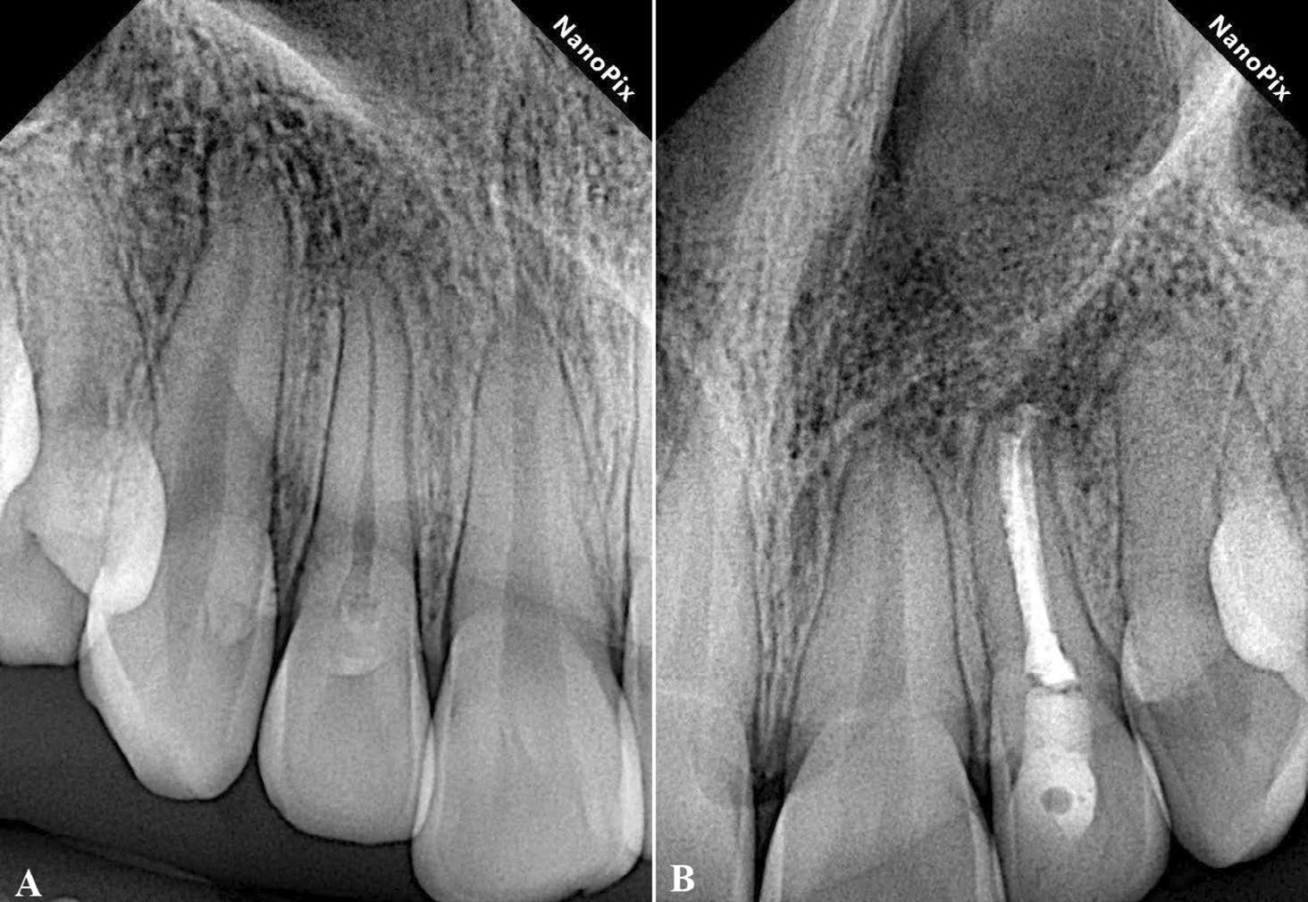
(A) The two‐year follow‐up radiographic image of the maxillary right lateral incisor (tooth #7) shows the effectiveness of the conservative restorative treatment approach in eliminating the inflammation and preventing its progression. (B) The two‐year follow‐up radiographic image of the maxillary left lateral incisor (tooth #10) shows significant healing of the periapical radiolucency and tissue regeneration with no evidence of inflammation.

The patient did not complain of any signs or symptoms at the six‐month follow‐up. Examinations revealed favorable healing of the bone and periapical lesion with reduced apical radiolucency for tooth #10 (Figure 6). As for tooth #7, the restoration remained intact, with no signs of caries or pulp involvement. After twenty‐four months of follow‐up, significant healing was observed in both teeth #7 and #10, confirming the success of the treatments for both teeth (Figure 7A,B).

## 6 Conclusions

Dens Invaginatus, a developmental malformation, predisposes teeth to pulp and periradicular diseases. The unique anatomy of a tooth with DI may be challenging in cases with pulp involvement and periapical inflammation that require endodontic treatments. A comprehensive approach involving clinical tests, periapical radiographic images, and CBCT is essential for accurate diagnosis and treatment planning. The results of this case report successfully demonstrated that, although managing DI may be challenging, with accurate diagnosis and appropriate treatment planning, successful outcomes are within reach.

## 7 Discussion

DI can be diagnosed through clinical and radiographic examinations. Depending on the severity of the malformation, the clinical manifestation of a tooth with DI can range from normal to irregular shapes [13, 17]. DI typically starts at the crown and may extend to the root, with or without communication with the surrounding tissues. The most common presentations include a bifid exaggerated cingulum or a conical shape [1, 2, 3].

Teeth with DI can go undetected during clinical examination in the absence of clinical signs or symptoms. Although in some cases, teeth with invagination may appear normal, they often display abnormal shapes [2]. DI is typically identified as an incidental radiographic finding unless the patient has pain or swelling associated with the involved tooth [18]. Therefore, it is recommended to radiographically examine abnormal teeth to check for DI or other malformations [1, 19].

DI typically appears as a radiolucent invagination surrounded by a radiopaque enamel and may associate with lateral or periapical radiolucency in case of extensive malformations [2]. However, conventional radiographs are often short on diagnostic information due to their limitations in exhibiting a two‐dimensional view of complex anatomy, especially in cases with lateral or periapical lesions [13]. In such cases, CBCT combined with clinical examination is helpful for diagnosis and treatment planning [10, 19, 20, 21].

The management of a tooth with DI depends on the extent of malformation, pulp and periapical involvement, and the stage of root development [12, 18]. Early detection and management of DI help prevent potential complications [19]. Based on these factors, treatment options can range from simple preventive sealing of invagination to non‐surgical root canal therapy, regenerative endodontic treatment, surgical procedures or even extraction [22].

In cases with Pulpoperiapical involvement requiring root canal therapy, the complex morphology of DI presents a significant endodontic challenge, especially in type II and III invaginations [9]. Another challenge associated with DI is the immature open apex of the involved teeth. DI increases teeth susceptibility to decay and bacterial infection, leading to early pulpal involvement and pulp necrosis, often before the root‐end closure [15, 18, 22]. This challenge was evident in the presented case, where the open apex was sealed with a bioceramic material as an apical barrier.

In the presented case, the teeth exhibited varying degrees of pulp and periapical involvement. The maxillary right lateral incisor (tooth #7) had an inflamed pulp with a healthy periodontium. When the pulp is vital, a conservative approach to seal the invagination is sufficient. In such cases, the invagination can be sealed with bioceramic materials or restorative materials like composite resin, which was the material of choice in this case [14, 15, 16].

The Maxillary left lateral incisor (tooth #10) was diagnosed with necrotic pulp and chronic apical abscess. Due to the presence of apical periodontitis and pulp necrosis, root canal treatment was indicated. Given the complex anatomy of the tooth, a combination of methods was used for successful treatment, including CBCT imaging, magnification with a dental microscope, and thermoplasticized obturation [1, 2]. The root of this tooth was immature, with an open apex that required sealing using either regenerative methods or apexification. In this case, the open apex was sealed with Cold Ceramic (CC) [23, 24, 25, 26].

The impact of early detection on the treatment plan is evident in this case report [18]. The maxillary right lateral incisor (tooth #7) was in the early stages of inflammation, which was managed with a conservative restorative treatment. Meanwhile, in the maxillary left lateral incisor (tooth #10), the bacterial infection and inflammation progressed to pulp necrosis, necessitating endodontic treatment. Had pulp involvement been detected earlier, tooth #10 also could have been managed with conservative approaches.

DI predisposes the tooth to bacterial infection and inflammation, which leads to pulp and periapical diseases [10, 11]. Early diagnosis of this anomaly and affected tooth simplifies the treatment process [7, 23]. In cases with extensive pulp or periapical involvement, a combination of methods must be used for effective treatment planning and management [9, 14, 27]. In the presented case, nonsurgical treatments, including resin restoration and root canal therapy, were effective in eliminating the infection and inflammation in the affected teeth, leading to bone formation and tissue healing. Additionally, a comprehensive understanding of the conditions and manifestations of DI is essential for developing an optimal treatment strategy and improving the prognosis for affected teeth [2, 10, 15].

## Author Contributions

**Ali Chamani:** conceptualization, investigation, resources, visualization, writing – review and editing. **Maryam Forghani:** conceptualization, supervision, validation, writing – review and editing. **Ghazal Asadi:** visualization, writing – original draft, writing – review and editing.

## Ethics Statement

For clinical cases, the local ethics committee considers that the patient's consent is sufficient.

## Consent

Written informed consent was obtained from the patient to publish this report in accordance with the journal's patient consent policy.

## Conflicts of Interest

The authors declare no conflicts of interest.

## Data Availability

The clinical pictures and radiographs data that support the findings of this study are included in the article.
